# Clinical outcome, recanalization success, and time metrics in drip-and-ship vs. drive-the-doctor: A retrospective analysis of the HEI-LU-Stroke registry

**DOI:** 10.3389/fneur.2023.1142983

**Published:** 2023-03-09

**Authors:** Christian Urbanek, Jasmin Jung, Resul Güney, Arne Potreck, Simon Nagel, Armin J. Grau, Timan Boujan, Andre Luckscheiter, Martin Bendszus, Markus A. Möhlenbruch, Fatih Seker

**Affiliations:** ^1^Department of Neurology, Clinical Centre of the City of Ludwigshafen, Ludwigshafen, Germany; ^2^Department of Neuroradiology, Heidelberg University Hospital, Heidelberg, Germany; ^3^Department of Neurology, Heidelberg University Hospital, Heidelberg, Germany; ^4^Department of Radiology, Clinical Centre of the City of Ludwigshafen, Ludwigshafen, Germany; ^5^Department of Anaesthesiology, Clinical Centre of the City of Ludwigshafen, Ludwigshafen, Germany

**Keywords:** stroke, thrombectomy, triage concept, drip and ship, drive the doctor

## Abstract

**Purpose:**

This study aimed at comparing clinical outcome, recanalization success and time metrics in the “drip and ship” (DS) vs. “drive the doctor” (DD) concept in a comparable setting.

**Methods:**

This is a retrospective analysis of thrombectomy registries of a comprehensive stroke center (CSC) and a thrombectomy-capable stroke center (TSC). Patients, who were transferred from the TSC to the CSC, were classified as DS. Patients treated at the TSC by an interventionalist transferred from the CSC were classified as DD. Good outcome was defined as mRS 0–2 or equivalent to premorbid mRS at discharge. Recanalization (TICI 2b-3 or equivalent) and time metrics were compared in both groups.

**Results:**

In total, 295 patients were included, of which 116 (39.3%) were treated in the DS concept and 179 (60.7%) in the DD concept. Good clinical outcome was similarly achieved in DS and DD (DS 25.0% vs. DD 31.3%, *P* = 0.293). mRS on discharge (DS median 4, DD median 4, *P* = 0.686), NIHSS improvement (DS median 4, DD median 5, *P* = 0.582) and NIHSS on discharge (DS median 9, DD median 7, *P* = 0.231) were similar in both groups. Successful reperfusion was achieved similarly in DS (75.9%) and DD as well (81.0%, *P* = 0.375). Time from onset to reperfusion (median DS 379 vs. DD 286 min, *P* = 0.076) and time from initial imaging to reperfusion were longer in DS compared to DD (median DS 246 vs. DD 162 min, *P* < 0.001).

**Conclusion:**

The DD concept is time saving while achieving similar clinical outcome and recanalization results.

## Introduction

Many studies have shown that timely initiation of thrombectomy in acute ischemic stroke is highly important as the odds of achieving good clinical outcome decrease over time (1–3). Therefore, stroke patient with large vessel occlusions need to be admitted quickly to a stroke center capable of performing thrombectomy. Efficient organization of regional stroke networks is challenging, though. Despite sufficient technical equipment and expertise in acute stroke care, not every tertiary care hospital is capable of performing thrombectomies at all times of the day and night, mainly due to a lack of interventional neuroradiologists (INR) or neurointerventionalists (4–6). Thrombectomy candidates at these hospitals therefore have to be transferred to a CSC for thrombectomy, which is known as the “drip and ship” (DS) concept. This interhospital transfer of stroke patients is associated with a relevant time loss, though (7–9).

To reduce this time loss, some stroke networks have initiated a concept in which INRs are transferred from a CSC to a thrombectomy-capable stroke center (TSC) to perform thrombectomy there instead of transferring these patients to a CSC (“drive the doctor” concept, DD). Several studies have shown that this concept can reduce time loss in the acute stroke treatment (10–18). Most of these studies had a complex referral system with various primary stroke centers and TSCs. Based on currently available data, a direct comparison of time metrics in DS and DD for a specific TSC is only possible to a limited extent.

The aim of this retrospective was to compare clinical outcome, recanalization success and time metrics in DS vs. DD within the setting of one university CSC and one community TSC (HEIdelberg LUdwigshafen Stroke cooperation, HEI-LU-Stroke).

## Materials and methods

### Study design and setting

**Figure 1:**
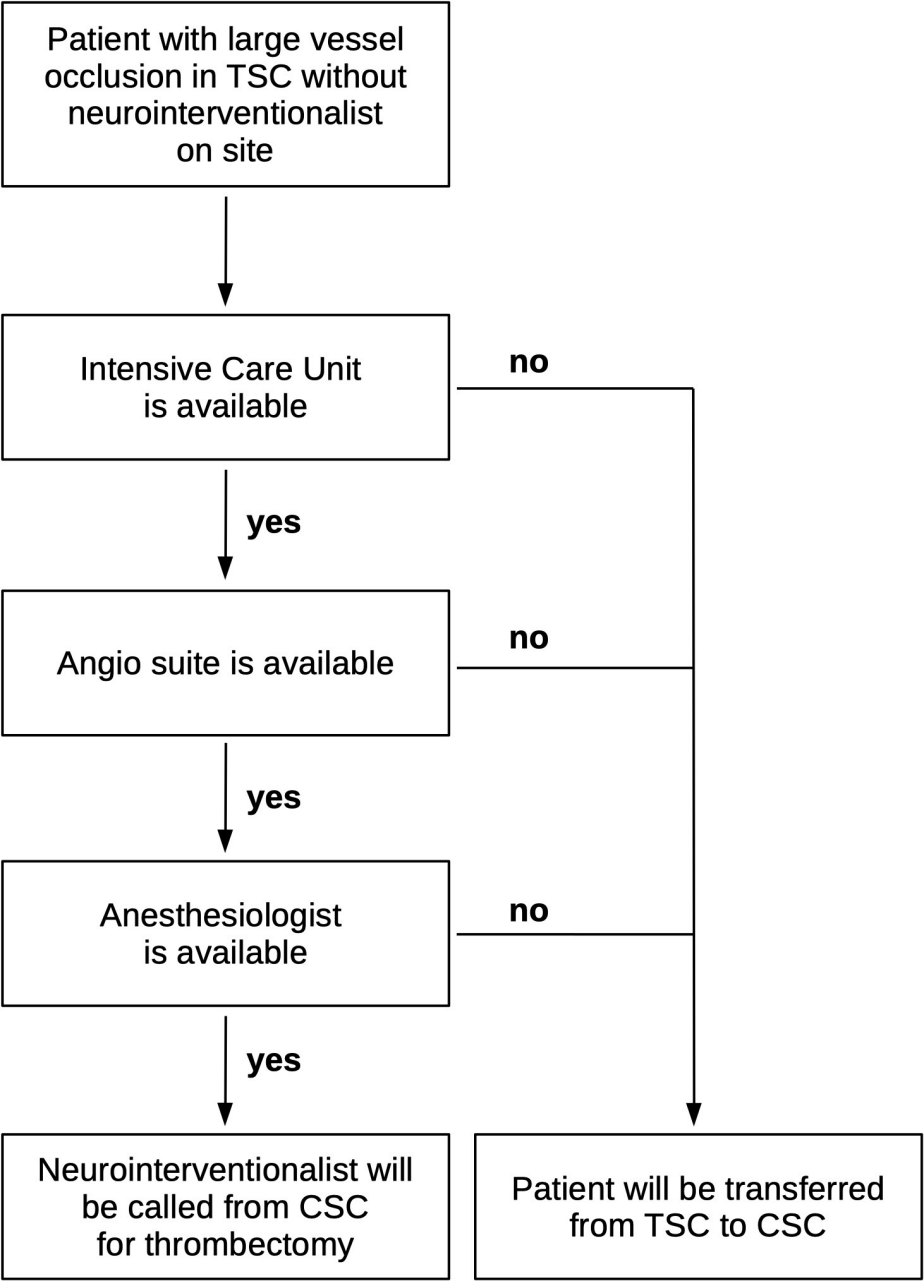
Flow chart of standards of operation at the thrombectomy-capable stroke center (TSC) and the comprehensive stroke center (CSC).

This is a retrospective observational bi-center study. Both centers are located in the southwest of Germany and about 25 km apart (driving time about 30 min). One of the centers is a university CSC in Heidelberg. Its INR team covers thrombectomy service at its CSC and at several TSCs in surrounding cities. The other center is a tertiary care community hospital in Ludwigshafen with many years of experience in acute stroke treatment and post-stroke care (TSC), but with a limited number of INRs. In order to offer a 24/7 thrombectomy service, this TSC made an agreement with the CSC. Briefly, whenever a thrombectomy candidate is admitted to the TSC, but no in-house INR is available, the neurologist on call at the TSC will check the availability of an anesthesiologist and an intensive care unit bed. If both are available, the CSC will be contacted and an INR will drive to the TSC by taxi or by car (DD concept). In the meantime, the interventional radiologist (without neurointerventional training) of the TSC is called as well and performs transfemoral puncture and placement of the sheath. If the TSC does not have capacity for a thrombectomy procedure, the patient will be transferred to the CSC (DS concept). Hence, the decision for DS or DD is not based on medical reasons, but only on the capacity of the TSC. The standards of operation are depicted in Figure 1. All patients were treated as per institutional standard of care.

Ethical committee approval was obtained. Informed consent was waived due to the retrospective study design. This manuscript is written according to the Strengthening the Reporting of Observational studies in Epidemiology guidelines.

### Inclusion and exclusion criteria

Each center recorded data of all stroke patients undergoing endovascular treatment in their institutional registries between January 2015 and December 2019. At the CSC, all patients who were transferred from the TSC and underwent endovascular treatment at the CSC were included (DS cohort). At the TSC, all patients who underwent endovascular treatment at the TSC, but performed by an INR from the CSC were included (DD cohort). No exclusion criteria were defined.

### Outcome measures

Primary clinical outcome measure was good clinical outcome, defined as mRS on discharge 0–2 or clinical recovery to the status before stroke onset (i.e., equal premorbid mRS and mRS and discharge). Ninety day mRS was not available for many patients treated at the TSC. Secondary clinical outcome measures were NIHSS on discharge and NIHSS improvement (NIHSS at discharge compared to NIHSS on admission).

Successful reperfusion was a radiological endpoint, which was defined as reperfusion of at least 50% of the territory of the target vessel (TICI 2b-3 or equivalent). Another endpoint was the occurrence of intraprocedural complications.

The following time measures were collected: Time from onset (or last seen well) to successful reperfusion (or end of procedure in case of failed reperfusion), time from initial imaging at the TSC to successful reperfusion, time from initial imaging to vascular puncture (i.e., begin of angiography), and time from puncture to reperfusion.

### Statistics

Statistical analysis was performed with R version 3.6.2 and RStudio version 1.2.5033 (RStudio, Boston, MA/USA). The non-parametric Whitney U-test and Fisher's exact test were used to assess differences in continuous and categorical variables. Univariate analysis was performed to identify potential predictors of good clinical outcome. All variables with *P* < 0.05 were then included in a multivariate analysis in order to identify independent predictors of good clinical outcome. A *P* value < 0.05 was considered statistically significant.

## Results

In total, 295 patients were included in this analysis of which 116 (39.3%) were treated in the DS concept and 179 (60.7%) were treated in the DD concept between January 2015 and December 2019. In the same period, 201 patients were treated at the TSC by its own INR team. Data of these patients are not available, though.

Baseline characteristics were similar in both cohorts. Hypertension was more frequent (91.6 vs. 77.6%, P < 0.001) and baseline NIHSS (median 15 vs. 16, *P* = 0.007) and premorbid mRS (median 0 in both groups, *P* = 0.014) were lower in the DD cohort. These differences reached statistical significance (Table 1).

**Table 1:** Demographics.

	**Drip and ship (*n* = 116)**	**Drive the doctor (*n* = 179)**	***P* value**
Age, years, mean (SD)	69.5 (15.9)	71.1 (13.0)	0.745
Female, *n* (%)	56 (48.3)	85 (47.5)	1.000
Hypertension, *n* (%)	90 (77.6)	164 (91.6)	0.002
Atrial fibrillation, *n* (%)	55 (47.4)	91 (50.8)	0.634
Diabetes, *n* (%)	25 (21.6)	35 (19.6)	0.767
Baseline NIHSS, median (IQR)	16 (13–22)	15 (10–19)	0.007
Premorbid mRS, median (IQR)	0 (0–2)	0 (0–1)	0.014
Occlusion site, *n* (%)			0.111
ICA	29 (25.0)	43 (24.0)	
MCA	65 (56.0)	122 (68.2)	
BA	19 (16.4)	13 (7.3)	
Other	3 (2.6)	1 (0.6)	
Intravenous thrombolysis, *n* (%)	82 (70.7)	121 (67.6)	0.609

BA, basilar artery; ICA, internal carotid artery; IQR, interquartile range; MCA, middle cerebral artery; mRS, modified Rankin Scale; NIHSS, National Institutes of Health Stroke Scale; SD, standard deviation.

Good clinical outcome was similarly achieved in DS and DD (DS 25.0 vs. DD 31.3%, *P* = 0.293). mRS on discharge (DS median 4, DD median 4, *P* = 0.686), NIHSS improvement (DS median 4, DD median 5, *P* = 0.582) and NIHSS on discharge (DS median 9, DD median 7, *P* = 0.231) were similar in both groups. Successful reperfusion was achieved similarly in DS (75.9%) and DD as well (81.0%, *P* = 0.375) (Table 2). Multivariate analysis revealed that DD is not a predictor of clinical outcome (Table 3).

**Table 2:** Outcome measures.

	**Drip and ship (*n* = 116)**	**Drive the doctor (*n* = 179)**	***P*-value**
Good clinical outcome, *n* (%)	29 (25.0)	56 (31.3)	0.293
mRS on discharge, median (IQR)	4 (3–5)	4 (2–5)	0.686
NIHSS improvement, median (IQR)	4 (−2–11)	5 (−3–11)	0.582
NIHSS on discharge, median (IQR)	9 (3–20)	7 (2–19)	0.231
Successful reperfusion, *n* (%)	88 (75.9)	145 (81.0)	0.375
Time onset to reperfusion, min	379 (295–492)	286 (232–425)	0.076
Time image to reperfusion, min	246 (204–307)	162 (130–214)	< 0.001
Time image to puncture, min	170 (135–209)	83 (65–104)	< 0.001
Time puncture to reperfusion, min	63 (39–116)	73 (54–114)	0.092

Time intervals are given as median and interquartile ranges.

**Table 3:** Multivariate analysis of good outcome at discharge.

	**Unadjusted OR (95% CI)**	***P-*value**	**Adjusted OR (95% CI)**	***P*-value**
Age (per year)	0.99 (0.97–1.01)	0.163		
Female	1.92 (1.15–3.23)	0.013^*^	1.72 (0.91–3.27)	0.096
**Comorbidities**				
Diabetes	0.55 (0.27–1.07)	0.091		
Hypertension	0.97 (0.48–2.08)	0.945		
Atrial fibrillation	0.97 (0.48–2.08)	0.945		
Premorbid mRS (per point)	0.74 (0.56–0.95)	0.022^*^	0.73 (0.52–0.99)	0.053
Baseline NIHSS (per point)	0.85 (0.8–0.89)	< 0.001^*^	0.81 (0.75–0.86)	< 0.001^*^
Intravenous thrombolysis	1.08 (0.63–1.90)	0.78		
Successful reperfusion	9.95 (3.53–41.67)	< 0.001^*^	11.22 (3.45–51.72)	< 0.001^*^
Onset to reperfusion (per 10 min)	0.99 (0.97–1.00)	0.048^*^	0.98 (0.97–1.00)	0.029^*^
Drive the doctor	1.37 (0.81–2.33)	0.245		

Asterisk indicates statistically significant P values.

**Figure 2:**
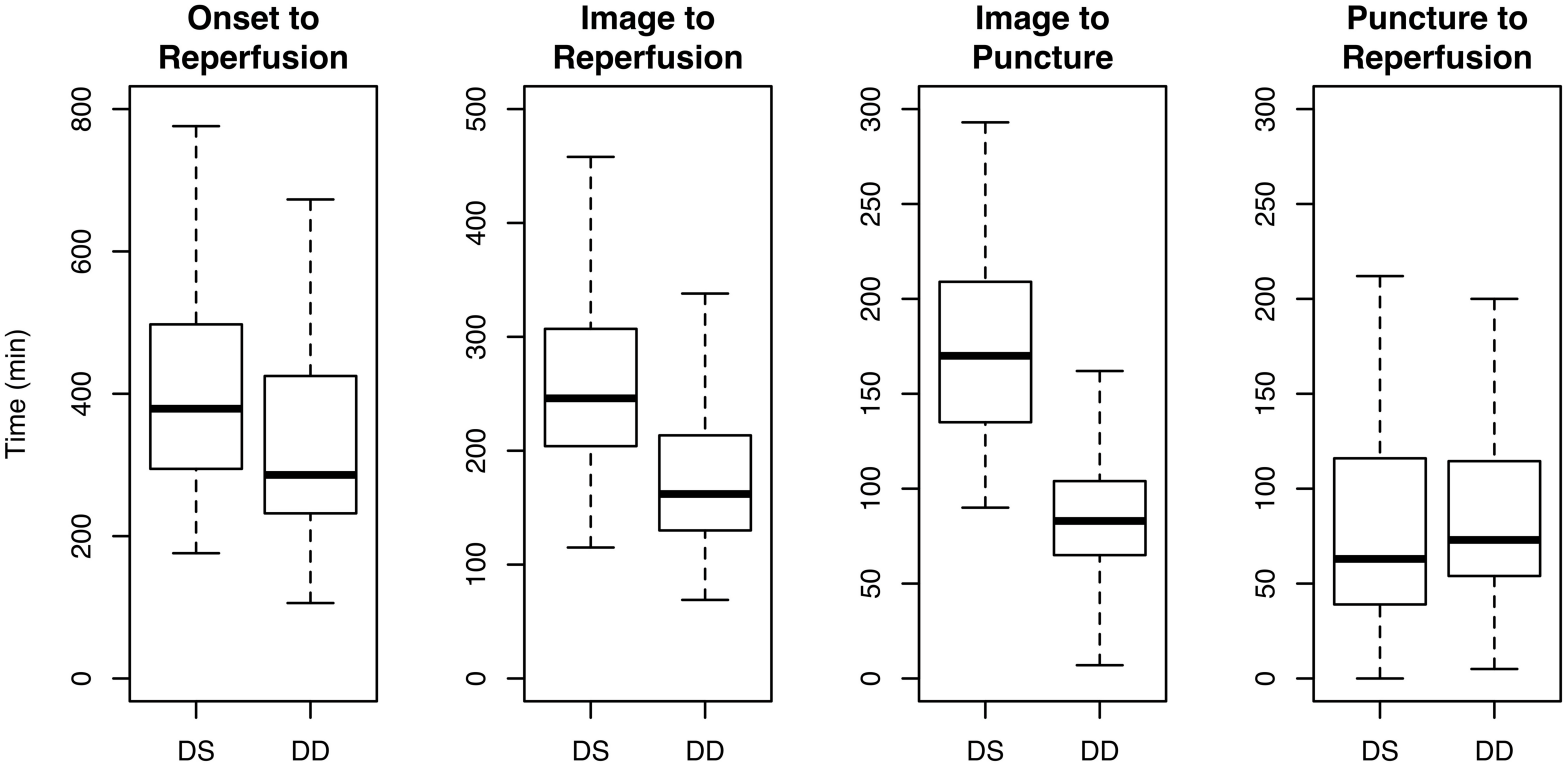
Time from onset to reperfusion, imaging to reperfusion, imaging to puncture and puncture to reperfusion in patients transferred from the thrombectomy-capable stroke center to the comprehensive stroke center (drip and ship, DS) vs. patients treated at the thrombectomy-capable stroke center by a neuroradiologist from the comprehensive stroke center (drive the doctor, DD).

Time from onset (or last seen well) to reperfusion was longer in DS compared to DD (median 379 vs. 286 min, *P* = 0.076). Time from initial imaging to reperfusion (or end of thrombectomy) was longer in DS compared to DD (median 246 vs. 162 min, P < 0.001). Time from imaging to groin puncture was longer in DS compared to DD (median 170 vs. 83 min, *P* < 0.001). Time from puncture to reperfusion was similar in both cohorts (median DS 63 min, DD 73 min, *P* = 0.093) (Table 2; Figure 2).

In 10 cases of the DD group (5.6%) and in 11 cases of the DS group (9.5%), intraprocedural complications occurred (*P* = 0.299). For instance, accidental dissections occurred in 2 cases in the DD and in 3 cases in the DS group, while perforations occurred in 4 cases in the DD and in 3 cases in the DS group.

## Discussion

Availability of neurointerventionalists for endovascular stroke treatment is limited, even in many tertiary care hospitals (4). Therefore, thrombectomy candidates at these hospitals have to be transferred to a CSC. Turning stroke centers, which meet certain requirements, to TSCs can be a solution for some hospitals (DD concept). Several studies on this concept under various names have been published in the recent years (10–16, 18). These studies mostly analyzed stroke networks with a complex referral system in which various primary stroke centers, CSCs and TSCs were involved. The present study is the largest one comparing the DS and DD concept between one CSC and one TSC.

According to our results, median time from initial stroke imaging to reperfusion was 84 mins shorter in DD compared to DS. This finding is consistent with results of previous studies (10, 15, 17). Interhospital transfer of stroke patients is known to be very time-consuming (7). In the DD concept, stroke patients can be prepared for thrombectomy in the angiography suite at the TSC, while the INR is transferred to the TSC. This parallelization saves time in the endovascular treatment of stroke.

In our study, the CSC and the TSC are located in different cities and the INRs either drove by car or were transferred by taxi. The usage of a helicopter, as done in a stroke network in Bavaria/Germany, can be an alternative, especially for long distances (18). However, it is more expensive than the usage of a car or a taxi. As shown by the Mobile Interventional Stroke Team in Manhattan, the DD concept is also feasible and time-saving in metropolises, which regularly deal with traffic congestion (12, 13, 17).

In our study, successful recanalization was achieved similarly in DS and DD and also similar to the results of the HERMES meta-analysis (19). The rate of intraprocedural complications was similar in both groups as well. Sufficient technical equipment is a requirement for adequate recanalization results and management of complications, because not every thrombectomy case can be handled with standard material (20). The TSC in the present study, for instance, has a fully equipped radiology department with various stent-retrievers, stents, wires, catheters, sheathes etc. and performs thrombectomies during working hours with its own INR team.

Excellent stroke treatment requires not only experienced INRs, but also experienced neurologists. The TSC in the present study has a neurology department with many years of experience in acute stroke treatment and post-stroke care. This is crucial for a quick recovery of stroke patients.

In accordance with previous studies (13, 14, 16, 18), clinical outcome results were similar in DS and DD. This may appear irritating as reperfusion was achieved faster in DD compared to DS. Saving of time could be demonstrated for the time frames “imaging to puncture” and “imaging to reperfusion.” “Onset to reperfusion” was shorter in DD compared to DS as well, however, without reaching statistical significance. Hence, the amount of time saving does not seem to be sufficient to have an impact on clinical outcome.

The DD concept has several advantages. It allows the TSC to provide 24/7 endovascular stroke treatment. It can also be beneficial for the CSC participating in this cooperation, as it disburdens its stroke unit and intensive care unit. This gives CSCs more capacity for elective cases and higher caseload for neurointerventional training.

However, the DD concept can be very stressful for INR teams depending on the caseload (21). Hence, the INR team of the CSC providing thrombectomy service at TSCs needs to be large enough. The Joint Commission has published suggestions for regional authorities concerning the inclusion of TSCs in stroke networks (22). We do not endorse the idea of regulatory bodies or hospital administrations forcing INR teams to cover TSCs. In the end, only INR departments can adequately judge whether they are able to cover another TSC. Mack et al. (23) also raised their concern about inadequate accreditation programs of the Joint Commission for TSCs.

This study has several limitations mainly due to the retrospective observational design. Patients were not randomly assigned to the DS or DD concept. Allocation to either concept depended mainly on the availability of an anesthesiologist and an intensive care unit bed. Nonetheless, groups were similarly matched regarding baseline characteristics. The generalizability of our findings may be limited, because a body-interventional radiologist (without neurointerventional experience) was present at the TSC and prepared the procedure. Still, our results showed comparable results to previous studies. Since 90 day mRS scores were not available for many patients treated at the TSC, mRS at discharge was used for outcome analysis. This might have led to a bias as the date of discharge was not documented. Nonetheless, early mRS has been reported to correlate strongly with mRS at 90 days (24). Future studies should address the impact on long-term clinical outcome. Generalizability of our results is also limited, because this study is based on a cooperation between two hospitals. Nonetheless, the study may provide useful data for hospitals that are interested in entering a similar cooperation. Future studies should also evaluate the cost effectiveness due to savings in e.g., patient transport, possible reimaging and reactivation of an emergency department at the CSC.

## Conclusion

DS is a wellestablished triage concept that will ensure access to thrombectomy for most stroke patients living in rural areas. Patients admitted to a sufficiently equipped stroke center, a so-called thrombectomy-capable stroke center, can be treated without hesitation at the TSC, if certain requirements are met. Within the setting of one CSC and one TSC, the DD concept is time saving while achieving similar results regarding recanalization success and clinical outcome compared to DS.

## Data availability statement

The original contributions presented in the study are included in the article/supplementary material, further inquiries can be directed to the corresponding author.

## Ethics statement

The studies involving human participants were reviewed and approved by Heidelberg University. Written informed consent for participation was not required for this study in accordance with the national legislation and the institutional requirements.

## Author contributions

Material preparation, data collection, and analysis were performed by CU, JJ, RG, AP, TB, AL, and FS. The first draft of the manuscript was written by FS. All authors contributed to the study conception and design, commented on previous versions of the manuscript, read, and approved the final manuscript.
